# Decision making and ambiguity in auditory stream segregation

**DOI:** 10.3389/fnins.2015.00266

**Published:** 2015-08-11

**Authors:** Susann Deike, Peter Heil, Martin Böckmann-Barthel, André Brechmann

**Affiliations:** ^1^Special Lab Non-invasive Brain Imaging, Leibniz Institute for NeurobiologyMagdeburg, Germany; ^2^Department of Systems Physiology of Learning, Leibniz Institute for NeurobiologyMagdeburg, Germany; ^3^Department of Experimental Audiology, Otto-von-Guericke-University MagdeburgMagdeburg, Germany

**Keywords:** auditory stream segregation, ambiguity, decision making, uncertainty, bistability

## Abstract

Researchers of auditory stream segregation have largely taken a bottom-up view on the link between physical stimulus parameters and the perceptual organization of sequences of ABAB sounds. However, in the majority of studies, researchers have relied on the reported decisions of the subjects regarding which of the predefined percepts (e.g., one stream or two streams) predominated when subjects listened to more or less ambiguous streaming sequences. When searching for neural mechanisms of stream segregation, it should be kept in mind that such decision processes may contribute to brain activation, as also suggested by recent human imaging data. The present study proposes that the uncertainty of a subject in making a decision about the perceptual organization of ambiguous streaming sequences may be reflected in the time required to make an initial decision. To this end, subjects had to decide on their current percept while listening to ABAB auditory streaming sequences. Each sequence had a duration of 30 s and was composed of A and B harmonic tone complexes differing in fundamental frequency (ΔF). Sequences with seven different ΔF were tested. We found that the initial decision time varied non-monotonically with ΔF and that it was significantly correlated with the degree of perceptual ambiguity defined from the proportions of time the subjects reported a one-stream or a two-stream percept subsequent to the first decision. This strong relation of the proposed measures of decision uncertainty and perceptual ambiguity should be taken into account when searching for neural correlates of auditory stream segregation.

## Introduction

Researchers of auditory stream segregation have largely taken a bottom-up view on the link between physical stimulus parameters and the perceptual organization of classical streaming sequences, i.e., series of, for example, high-frequency A and low-frequency B tones presented in alternation (ABAB) or as repeated ABA triplets (ABA_; where _ represents a pause) (Van Noorden, 1975). However, in the majority of studies, researchers have relied on the reported decisions of subjects regarding which of two or more predefined percepts (e.g., that of a single stream or that of two separated A and B streams) predominates when listening to such streaming sequences (for a recent review, see Winkler et al., 2012). Recent imaging data from humans (Dollezal et al., 2014) suggest an involvement in such tasks of brain regions which are more generally involved in decision processes. When subjects listened to the streaming sequences of the highest ambiguity, i.e., those that led to roughly equal probabilities of the one-stream and the two-stream percept, Dollezal et al. (2014) found stronger BOLD signals in the left posterior medial frontal gyrus (pMFG) and the left posterior cingulate gyrus (PCG) than when subjects listened to sequences where one of the perceptual alternatives dominated. Both regions have been associated with cognitive functions, monitoring response conflicts and decision uncertainty (Ridderinkhof et al., 2004) and being involved when higher task demands were imposed (Raichle et al., 2001; Dosenbach et al., 2007), respectively. This suggests that perceptual ambiguity is associated with an uncertainty to decide for the appropriate perceptual organization and with a higher cognitive load due to this uncertainty. Any brain activation elicited by such processes may obscure activation elicited by the streaming sequences itself and, thus, renders the search for the neural mechanisms of stream segregation more difficult. The present study aims at distinguishing the two processes by assessing independent behavioral correlates of decision uncertainty and perceptual ambiguity in auditory stream segregation.

In cognitive psychological research, decision processes in perceptual classification tasks are quantified by the response time (Donders, 1969; Maddox et al., 1998; Nosofsky and Stanton, 2005; Palmer et al., 2005; Ratcliff and Mckoon, 2008; Seger and Peterson, 2013). Specifically, the response time has been shown to increase with task difficulty, i.e., with decreasing perceptual certainty and with decreasing stimulus strength. We therefore adopt the time taken by the subject to decide on, and report, the initial percept (*initial decision time*) as a measure of the decision uncertainty in the task of classifying streaming sequences and investigate its relation to the perceptual ambiguity of such sequences.

There is currently no agreed-upon criterion to quantify perceptual ambiguity in stream segregation. Here we introduce an ambiguity index that quantifies perceptual ambiguity based on the proportions of time over which the perceptual alternatives occur, using previous behavioral data (Deike et al., 2012). In that study, we argued that the time needed to come to a decision about the initial percept of a streaming sequence should be excluded from calculating the proportions of a one-stream or a two-stream percept. We now suggest to use this initial response time as a measure of decision uncertainty and we investigate its relation to the ambiguity index.

Other behavioral measures of stream segregation, specifically perceptual phase duration and switching rate, have also been analyzed in the context of stream segregation, however, mainly to qualitatively describe the bistability or the temporal dynamics of perceptual organizations in ambiguous streaming sequences (Pressnitzer and Hupé, 2006; Denham et al., 2013). Perceptual bistability of ambiguous auditory streaming sequences as well as ambiguous visual figures is characterized by three fundamental aspects, i.e., exclusivity, inevitability, and randomness (Leopold and Logothetis, 1999; Pressnitzer and Hupé, 2006). In short, this means that the different perceptual interpretations can never occur simultaneously (exclusivity) and that, notwithstanding the possibility of volitional control, switches between perceptual interpretations occur unavoidably (inevitability) and at randomly distributed times (randomness). The two common measures of bistability, perceptual phase duration and switching rate, provide additional information to percept proportions because a given proportion can be achieved by many short phases (i.e., many switches) or by few long phases (i.e., no or few switches). We also assess these measures and test their relation to the degree of perceptual ambiguity. We argue that, when searching for the neural basis of streaming, it is important to differentiate between the phenomena of decision uncertainty, perceptual ambiguity, and stability of perceptual organization, because they may recruit different neural mechanisms. As a first step, the present study aims at (1) identifying independent behavioral correlates of these specified phenomena and (2) characterizing the relation of perceptual ambiguity to decision uncertainty and perceptual stability in auditory stream segregation.

## Materials and methods

The present study is based on the same experiments conducted for a previous study (Deike et al., 2012), where more details can be found. Here, we perform new analyses of these data with respect to the topics of decision uncertainty and bistability.

### Subjects

Twenty-two listeners (9 male, 13 female), aged between 19 and 38 years (mean age 27 years), participated in the experiments. Nine of them had enjoyed special musical training for 2–5 years during their childhood. All subjects had normal audiograms, with absolute thresholds ≤20 dB hearing level. The subjects gave written informed consent to the study which was approved by the Ethics Committee of the Otto-von-Guericke University of Magdeburg.

### Apparatus, stimuli, and procedure

**Figure 1:**
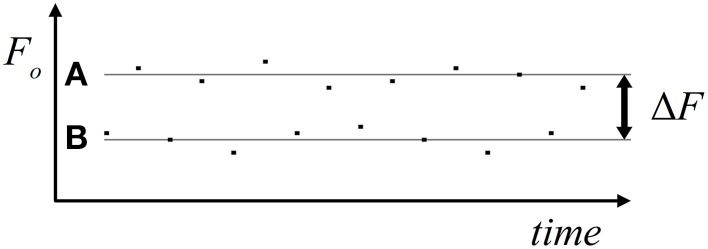
**Stimulus material**. The schematic depiction shows, as an example, part of a sequence of the ΔF condition of 10 semitones. Squares represent the A and B tone complexes. The geometric means of their F_0_ (gray lines) differ by 10 semitones (logarithmic scale). The F_0_ of the individual exemplars of A and B tone complexes differ from their geometric means by 0, ±1, or ±2 semitones.

The psychophysical measurements were performed in an acoustically shielded chamber (Industrial Acoustic Company, Niederkrüchten, Germany). The stimuli, which were digitally synthesized in Matlab (The Mathworks Inc., Natick MA, USA), were harmonic tone complexes comprising the fundamental frequency, F_0_, and four partials with frequencies from 2 F_0_ to 5 F_0_. All partials started and ended simultaneously and had equal amplitude. Each tone complex lasted 25 ms including 3.8 ms cosine-squared onset and offset ramps. The tone complexes were presented in ABAB sequences of 30 s duration with a presentation rate of 6 Hz. A and B tone complexes covered different F_0_ranges. In different conditions, seven average frequency separations (ΔF) between the F_0_ of A and B tone complexes were used, viz., 2, 4, 6, 8, 10, 12, and 14 semitones. These ΔF-values were achieved by varying the F_0_ of both the A and B tone complexes between conditions and relative to a F_0_ center of 392 Hz. In this way, the subjects were prevented from getting familiar with a specific frequency, which might have biased their percept toward the two-stream option. In addition, within each condition, individual exemplars of both A and B tone complexes varied in F_0_, differing from the geometric mean by 0, ±1, or ±2 semitones (F_0_ variants). Within sequences, the different F_0_ variants were presented randomly and with equal probability. The assigned ΔF-values therefore represent the geometric mean F_0_ separations between A and B tone complexes. Part of an example sequence (ΔF of 10 semitones) is schematically depicted in Figure 1. For each of the seven ΔF conditions, five different random sequences of A and B tone complexes were generated and used to prevent subjects from getting familiar with a specific sequence, which again might have biased the percept toward one or the other perceptual organization. Each random sequence was presented twice resulting in the presentation of 10 sequences per ΔF condition during the experiment. The different sequences were presented in pseudo-random order and alternated with silence of 10 s duration. The stimuli were presented binaurally via headphones (Sennheiser, HD 465) at an individually adjusted, comfortable sound level, using Presentation (Neurobehavioral Systems Inc., San Francisco).

Prior to the psychophysical measurements, the subjects received written instructions and additional verbal explanations if necessary. The subjects were asked to listen to the sound sequences and to continuously indicate their current percept by pressing the left mouse button with their right index finger when they perceived the low-F_0_ and high-F_0_ tone complexes as one coherent stream, and the right mouse button with their right middle finger when they perceived them as two separate streams, i.e., when they heard a low and a high stream in parallel. The subjects were encouraged to indicate as promptly as possible after the onset of each sequence whether they heard one stream or two streams and to update their response every time the percept switched, until the end of the sequence. The type of all button presses and their timing relative to sequence onset were recorded. All subjects performed the experiment twice on two different days. To familiarize the subjects with the sound sequences and the task, they were exposed to two sequences prior to the actual measurements. The two familiarizing sequences employed the 2 and the 14 semitone ΔF conditions, which are most likely to promote one or the other perceptual alternative, i.e., the one-stream and the two-stream percept, respectively.

### Data analysis

As a measure of the perceptual ambiguity, we computed an ambiguity index for each subject and each ΔF condition, across all 20 sequence presentations from both measurements. The ambiguity index is defined as:

$$ambiguity\;index\;=\;1-\left(\left|(P_{1}-P_{2})/(P_{1}+P_{2})\right|\right)$$

where *P*_1_ and *P*_2_ denote the probabilities of the one-stream and the two-stream percept. These probabilities are defined as the proportions of time the sequences were perceived as one stream or two streams, respectively (see Deike et al., 2012). For the calculation of the proportions, all phases following the first decision up to the end of the sequence were included. The ambiguity index can take on values between 0 and 1, with 1 indicating the strongest ambiguity, i.e., evenly balanced percept probabilities, and 0 indicating the complete dominance of one perceptual alternative.

To determine the certainty of the perceptual classification of the streaming sequences we considered the initial decision time as a cognitive performance measure for decision uncertainty (Maddox et al., 1998; Nosofsky and Stanton, 2005; Palmer et al., 2005; Ratcliff and Mckoon, 2008; Seger and Peterson, 2013). Furthermore, to determine the bistable characteristics of the sequences we considered the switching rate per sequence and the duration of the first perceptual phase as measures that are commonly used to describe bistability (Hupé and Rubin, 2003; Pressnitzer and Hupé, 2006; Denham et al., 2013). Note that the duration of the first perceptual phase was limited either by another button press indicating a perceptual change after the first decision or by the end of the sequence (when the first perceptual decision held to the end of the 30 s sequence). The decision uncertainty and bistability measures were calculated for each subject and each ΔF condition, averaged across all 20 sequence presentations from both measurements. The resulting values of each measure across the 22 subjects and the seven ΔF conditions showed skewed distributions. Therefore, a non-parametric statistical analysis was performed and diagrams depict the median and interquartile ranges of the corresponding data.

To examine the relationship of the considered decision uncertainty and bistability measures to the ambiguity index Spearman's correlations (coefficient ρ) were computed across the 22 subjects and the seven ΔF conditions, resulting in a sample size of *n* = 154. Significance levels were corrected for multiple comparisons using Bonferroni's correction (*p* = 0.05∕3 = 0.017).

## Results

**Figure 2:**
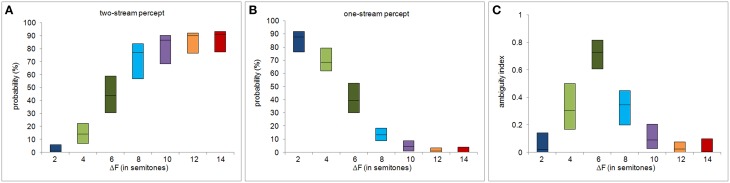
**Percept probabilities and ambiguity index**. The panels show, as functions of ΔF, the proportions of time that the stimulus sequences were perceived as two streams **(A)** and as one stream **(B)**, and the resulting ambiguity index **(C)**, defined on the percept probabilities shown in **(A,B)**. The central black line of each box represents the median and the upper and lower edges of the boxes the 25th and the 75th percentiles.

**Figure 3:**
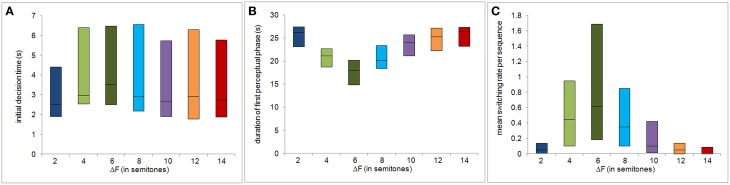
**Decision uncertainty and bistability measures as a function of the frequency separation ΔF**. The panels show, as functions of ΔF, the initial decision time **(A)**, the duration of first perceptual phase **(B)**, and the mean switching rate per sequence **(C)**. The central black line of each box represents the median and the upper and lower edges of the boxes the 25th and the 75th percentiles.

Figures 2A,B show *P*_1_ and *P*_2_ as functions of the frequency separation between the A and B tone complexes, ΔF, taken from Deike et al. (2012). Note that the summed proportions do not add up to 100%. The “missing” proportion corresponds to the time between sequence onset and the initial decision. From the percept probabilities subsequent to the initial decision, we computed the ambiguity index for each subject and each ΔF condition. The variation of the ambiguity index with ΔF is shown in Figure 2C. Figures 3A–C show the other behavioral measures as functions of ΔF. All measures vary non-monotonically with ΔF, showing either a maximum or a minimum at the ΔF condition of six semitones. Non-parametric Friedman tests for related samples reveal that all behavioral measures vary significantly with ΔF [ambiguity index: χ^2^(6) = 82.462, *p* < 0.0001; initial decision time: χ^2^(6) = 45.857, *p* < 0.0001; duration of first perceptual phase: χ^2^(6) = 74.708, *p* < 0.0001; mean switching rate per sequence: χ^2^(6) = 64.274, *p* < 0.0001].

Notably, the variability of the decision uncertainty and bistability measures also depends on ΔF and for perceptual phase duration and switching rate is highest in the ΔF condition with the highest ambiguity (see Figures 3A–C). Some of this variability might be due to variability of the decision criteria between subjects. It should be noted that the background of the subjects' experience was diverse. In particular, 9 of the 22 participants had received special musical training for 2–5 years during their childhood. The ability to follow one of the instruments playing in an ensemble represents an important musical capacity and might influence stream segregation (Marozeau et al., 2010). Thus, the musical background might have an effect on the inclination of subjects to switch their percept. However, high switching rates were found among participants with and without musical background. Therefore, no evidence for an effect of musical experience was found.

**Figure 4:**
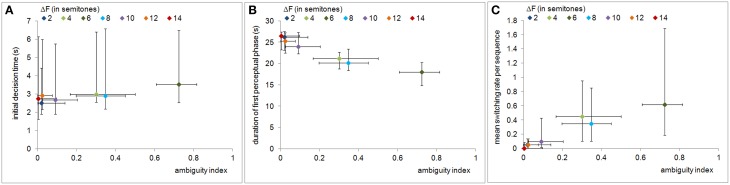
**Relation of perceptual ambiguity to decision uncertainty and bistability measures**. The panels show, as functions of the ambiguity index, the initial decision time **(A)**, the duration of the first perceptual phase **(B)**, and the mean switching rate **(C)**. Symbols and error bars represent medians and the 25th and 75th percentiles, respectively. All behavioral measures are significantly correlated with the ambiguity index.

The Spearman's correlations reveal that the ambiguity index was significantly correlated with the measure of decision uncertainty, i.e., the initial decision time (ρ = 0.342, *p* < 0.01), as well as with the measures of bistability, i.e., the duration of the first perceptual phase (ρ = −0.703, *p* < 0.01) and the mean switching rate per sequence (ρ = 0.475, *p* < 0.01). Figures 4A–C illustrate the relations of all behavioral measures to the ambiguity index values. The duration of the first perceptual phase decreased whereas the two other behavioral measures increased as the ambiguity of the ABAB stimulus sequences increased.

## Discussion

The present study aimed at characterizing the relation between perceptual ambiguity and decision uncertainty. To this end, ambiguity was quantified by an ambiguity index calculated from the respective probabilities of the two perceptual organizations. This index was found to be maximal at an intermediate ΔF-value of six semitones (Figure 2C). The calculation of the ambiguity index did not include the initial decision time. Instead, this response time was utilized separately as a measure of decision uncertainty. These two measures were significantly correlated. Thus, in imaging experiments that use streaming tasks and that rely on the reported decisions of the subjects regarding two predefined percepts (one-stream vs. two-stream percept), any observed brain activation may be explained by perceptual processes but also by decision processes.

As far as we are aware, up to now there are two human imaging studies that provide evidence for the influence of higher-order cognitive processes on the processing of ambiguous streaming sequences. Dollezal et al. (see bottom row of Figure 5 in Dollezal et al., 2014) observed in left PCG a stronger deactivation for the most ambiguous ABA_ streaming sequences. A stronger deactivation of this region is thought to reflect an increased attentional demand due to increased task difficulty (Raichle et al., 2001; Dosenbach et al., 2007). In addition, Dollezal et al. (see bottom row of Figure 8 in Dollezal et al., 2014) also found a stronger activation for the most ambiguous streaming sequences in the posterior medial frontal cortex, a region that is associated with the monitoring of response conflicts and decision uncertainty (Ridderinkhof et al., 2004). In auditory cortex, no effect of decision uncertainty was observed in the BOLD response data of Dollezal et al. (2014). However, in a recent magnetoencephalography study, Gutschalk et al. (2015) showed an enhanced peak-to-peak amplitude of the P1m-N1m complex of the auditory evoked magnetic fields for ambiguous but not for non-ambiguous ΔF conditions.

Supporting evidence for the influence of higher-order cognitive processing on the perception of ambiguous stimuli and specifically that of perceptual decision making comes from the visual domain. Here sensory and higher-order areas in parietal and frontal lobe have been shown to interact in determining perceptual decisions on ambiguous figures (for recent review see, Kleinschmidt et al., 2012). Taken together, these neural data complement our present behavioral findings suggesting a potential influence of higher-order cognitive processing on the perceptual processing of ambiguous streaming sequences in auditory cortex and beyond. This poses a problem when searching for the neural correlates of streaming.

On the other hand, the correlation between the ambiguity index and the initial decision time may be exploited in psychoacoustic experiments that aim at identifying the ambiguity of streaming sequences. The advantage could be that it may suffice to use only short sequences, of the order of the longest times to first decision (about 7 s in our case, Figure 3A), and only rely on the initial decision times for the classification of ambiguity.

The two measures of bistability, i.e., the switching rate and the first phase duration, were both significantly correlated with the perceptual ambiguity. However, regarding the temporal dynamics of streaming, the ΔF conditions which resulted in ambiguity indices < 0.1 (ΔF of 2, 10, 12, 14 semitones) do not provide much information, since the first decision on the perceptual organization is most often maintained for the remaining time so that a switch occurs in very few blocks only. Using somewhat different stimulus parameters from the present ones (pure tones instead of harmonic complex tones, tone duration of 120 ms instead of 25 ms, presentation rate of 8.3 Hz instead of 6 Hz, sequence duration of 4 min instead of 30 s), Pressnitzer and Hupé (2006) found a probability of 50% for both perceptual alternatives for a ΔF of five semitones. This probability suggests an ambiguity index close to 1. They reported a duration of about 20 s for the first perceptual phase which corresponds well to the phase durations found in the present data in the most ambiguous condition (ΔF of six semitones). Thus, in highly ambiguous sequences it seems to take about 20 s on average to switch to the alternative percept, a duration that needs to be confirmed with different parameter values and paradigms. To this end, the ambiguity index proposed here may provide a useful tool to compare bistability measures across the different experiments. Because previous studies reported either proportions of perceptual organization (e.g., Pressnitzer and Hupé, 2006; Bendixen et al., 2010) or switching rates only (Denham et al., 2013) but never both variables, quantitative comparison of the existing data is difficult. We suggest the ambiguity index as an appropriate measure to assess the ambiguity of a sequence across different future studies.

According to Pressnitzer and Hupé (2006) perceptual switches most probably occurred inevitably because of the subjects' tasks of monitoring but not volitionally controlling the perception. However, because both the switching rate and the measure of uncertainty—which we interpret as a top-down process—correlate similarly well with the ambiguity index, it is difficult to disentangle the bottom-up and top-down influences on streaming. Thus, we would agree with Leopold and Logothetis (1999) and Long and Toppino (2004) who suggest that both bottom-up and higher-order top-down processes govern multi-stable phenomena. In this view the brain acts in a way to find all possible interpretations of the sensory input, and perceptual changes occur “when stimuli are truly ambiguous and perception can never become ‘locked' onto a single solution” (Leopold and Logothetis, 1999, p. 261). According to this, all stimulus sequences tested in the present study are ambiguous because perceptual switches occurred even in the ΔF conditions where one perceptual organization dominated, which is in accordance with the findings of other studies (Denham and Winkler, 2006; Kashino et al., 2007; Rahne et al., 2008; Denham et al., 2013).

The occurrence of perceptual switches over such a broad parameter range might just be a consequence of the “artificial” experimental setting. The problem in such settings is that the subject cannot verify whether a given perceptual organization is reasonable or meaningful. In contrast, in real world situations, such verification is possible because sounds originate from certain sound sources which allow gathering additional information to disambiguate the auditory percept. The absence of an external proof in experimental settings makes it impossible to find the “correct” perceptual organization (because all are correct) and, thus, finally causes an uncertainty in perceptual decision.

## Funding

This work was supported by the “Deutsche Forschungsgemeinschaft” [SFB/TRR31].

### Conflict of interest statement

The authors declare that the research was conducted in the absence of any commercial or financial relationships that could be construed as a potential conflict of interest.
